# The relationship of *Megamonas* species with nonalcoholic fatty liver disease in children and adolescents revealed by metagenomics of gut microbiota

**DOI:** 10.1038/s41598-022-25140-2

**Published:** 2022-12-20

**Authors:** Jianli Zhou, Qiao Zhang, Yuzhen Zhao, Yu Zou, Moxian Chen, Shaoming Zhou, Zhaoxia Wang

**Affiliations:** 1 Division of Gastroenterology, Shenzhen Children’s Hospital, Shenzhen, 518000 Guangdong China; 2 Key Laboratory of National Forestry and Grassland Administration on Subtropical Forest Biodiversity Conservation, Co-Innovation Center for Sustainable Forestry in Southern China, College of Biology and the Environment, Nanjing Forestry University, Nanjing, 210037 China

**Keywords:** Microbiology, Medical research

## Abstract

Nonalcoholic fatty liver disease (NAFLD) is the most common chronic liver disease in children and adolescents. The gut microbiota plays an important role in the pathophysiology of NAFLD through the gut–liver axis. Therefore, we aimed to investigate the genus and species of gut microbiota and their functions in children and adolescents with NAFLD. From May 2017 to July 2018, a total of 58 children and adolescents, including 27 abnormal weight (AW) (obese) NAFLD patients, 16 AW non-NAFLD children, and 15 healthy children, were enrolled in this study at Shenzhen Children’s Hospital. All of them underwent magnetic resonance spectroscopy (MRS) to quantify the liver fat fraction. Stool samples were collected and analysed with metagenomics. According to body mass index (BMI) and MRS proton density fat fraction (MRS-PDFF), we divided the participants into BMI groups, including the AW group (n = 43) and the Lean group (n = 15); MRS groups, including the NAFLD group (n = 27) and the Control group (n = 31); and BMI-MRS 3 groups, including NAFLD_AW (AW children with NAFLD) (n = 27), Ctrl_AW (n = 16) (AW children without NAFLD) and Ctrl_Lean (n = 15). There was no difference in sex or age among those groups (*p* > 0.05). In the BMI groups, at the genus level, *Dialister*, *Akkermansia*, *Odoribacter,* and *Alistipes* exhibited a significant decrease in AW children compared with the Lean group. At the species level, *Megamonas hypermegale* was increased in the AW group, while *Akkermansia muciniphila*, *Dialister invisus*, *Alistipes putredinis*, *Bacteroides massiliensis*, *Odoribacter splanchnicus,* and *Bacteroides thetaiotaomicron* were decreased in AW children, compared to the Lean group. Compared with the Control group, the genus *Megamonas*, the species of *Megamonas hypermegale* and *Megamonas rupellensis,* increased in the NAFLD group. Furthermore, the genus *Megamonas* was enriched in the NAFLD_AW group, while *Odoribacter*, *Alistipes*, *Dialister,* and *Akkermansia* were depleted compared with the Ctrl_Lean or Ctrl_AW group at the genus level. *Megamonas hypermegale* and *Megamonas rupellensis* exhibited a significant increase in NAFLD_AW children compared with the Ctrl_Lean or Ctrl_AW group at the species level. Compared with healthy children, the pathways of P461-PWY contributed by the genus *Megamonas* were significantly increased in NAFLD_AW. We found that compared to healthy children, the genus *Megamonas* was enriched, while *Megamonas hypermegale* and *Megamonas rupellensis* were enriched at the species level in children and adolescents with NAFLD. This indicates that the NAFLD status and/or diet associated with NAFLD patients might lead to the enrichment of the genus *Megamonas* or *Megamonas* species.

## Introduction

Nonalcoholic fatty liver disease (NAFLD) is the most common cause of chronic liver disease in children and adolescents and is the second most common reason for liver transplantation in the world^1,2^. It includes a wide range of liver pathologies, from steatosis to nonalcoholic steatohepatitis (NASH). Currently, approximately 25% of the global population has NAFLD, with the highest prevalence in South America and the Middle East and the lowest prevalence in Africa^3^. Approximately 2.6–11.3% of children and approximately 40–70% of obese children are diagnosed with NAFLD worldwide^4^. In China, approximately 2% of children and 68% of obese children have NAFLD^5^. As a consequence, NAFLD is a major public health problem that needs more attention from children and adolescents.

The human microbiome includes bacteria, archaea, fungi, protozoans, and viruses, which have the same number of genes as 100 times more genes than the human body^6,7^. The genes encode a group of pathways that produce bioactive molecules derived from diet or metabolism^8^. However, the gut microbiota has many beneficial functions, such as obtaining energy from indigestible dietary fiber, which links the microbiota and its metabolites to the development of diseases, including obesity, NAFLD, and other diseases^9^.

When compared with healthy children, the gut microbiota of obese and NASH children showed a significant increase in *Bacteroides* and a significant decrease in *Firmicutes*^10^. In a study of children, a low abundance of *Oscillospira* with high levels of 2-butanone was characteristic of intestinal microbiota in hepatic steatosis, while the high abundances of *Ruminococcus* and *Dorea* were associated with the progression from steatosis to NASH^11^. Other studies showed that the α-diversity of gut microbiota in NAFLD of children was lower than in non-NAFLD obese children^12^. Recently, characteristic changes in gut microbiota may be associated with the pathogenesis of NAFLD and may be used as markers of the disease.

Although mouse models have revealed the pathophysiological processes of gut microbiota in NAFLD, evidence in children is limited^13^. Several studies have reported changes in the fecal microbiota in NAFLD in children, but these studies were small and lacked best-characterized cases and controls. Therefore, we investigated the gut microbiota of children with NAFLD, abnormal weight (AW) (obese) children without NAFLD, and healthy children to characterize the potential differences in the composition of their gut microbiota and functional capacity among them.

## Material and methods

### Study subjects

This study was approved by the Human Ethics Committee of Shenzhen Children’s Hospital (Permission No. 2016020). These subjects came from our team's previous research, and we re-analyzed the original data^14^. Participants aged from 10 to 17 years old (average age of 13.7 ± 1.8 years) were enrolled, including 43 abnormal weight (AW) (obese) children and adolescents [body mass index (BMI) above age- and gender-specific 95th percentile] and 15 healthy controls from May 2017 to July 2018 at Shenzhen Children’s Hospital (detailed in Table S1). According to a previous study, they all underwent single-voxel MRS scanning using a 3.0 T MR unit (MAGNETOM Skyra, Siemens Healthcare, Erlangen, Germany)^15^. NAFLD was diagnosed on the basis of MRS proton density fat fraction (MRS-PDFF) > 5%. Children who recently (past 90 days) used probiotics or antibiotics were excluded.

### Sample collection and metagenomic sequencing

Before the test, fecal samples were collected using a sterile kit and immediately stored in a − 80 °C refrigerator. DNA was isolated from fecal samples, yielding 343 Gb(mean 5.92 Gb) of sequence from each participant by the Beijing Genomics Institute (BGI, Shenzhen, China), where the following parts were analysed. All of the DNA fragments were purified using the QIAquick PCR Purification Kit (Qiagen) during library construction. The sample libraries were quantified by the ABI StepOnePlus Real-Time PCR System and Agilent 2100 Bioanalyzer. Then, the Illumina HiSeq System (Illumina HiSeq 2500, 4000, Xten and Novaseq) was used to sequence for qualified libraries.

### Metagenomic analysis

First, quality control (QC) of FASTQ^16^ files was used to filter the low-quality reads (average of 8% of each sample). Second, we applied SOAP2.2^17^ to remove reads (average of 0.1% of every sample) with 90% similarity to the human genome to acquire 284 Gb of clean reads (average of 4.9 Gb) (detailed in Table S2). Then, the clean reads were used for taxonomic annotations of each sample using MetaPhlAn2^18^. We removed the abundance < 0.01% of annotations, retained the bacteria and archaea to normalize, removed the unclassified levels and noname, and retained the taxonomic compositions, which were present in > 20% of samples (40 genera and 93 species) (detailed in Table S3 and Table S4). Next, we used HUMAnN2^19^ for functional annotations and removed microbial pathways with relative abundances < 0.01% and low presence (≤ 20%) in the total cohort (detailed in Table S7). We confirm that all methods were carried out in accordance with relevant guidelines and regulations.

### Statistical analysis

The data were analysed and visualized using R 3.5.1 with the ‘vegan’, ‘stats’ and ‘ggplot2’ packages. Numerical variables were tested for normality using the Shapiro test. The Wilcoxon rank-sum test was conducted to test the differences in age, BMI and MRS-PDFF. The chi-square test was performed to test the differences in gender distribution between groups. Shannon index was used to calculate the alpha diversity. Bray‒Curtis dissimilarity at the genus level was calculated for beta diversity. Diversity and differential abundance analysis of microbial traits between each pair of groups was performed by the Wilcoxon rank-sum test. Principal coordinates analysis (PCoA) was conducted based on the Bray‒Curtis distance at the genus level. Permutational multivariate analysis of variance (PERMANOVA) based on the above genus-level distance matrices was performed to test if an overall difference in microbial composition existed between groups (999 permutations). Spearman’s rank correlation analysis was conducted to assess the correlations between host phenotypes and the relative abundances of gut microbial features. A Benjamini‒Hochberg (BH)-adjusted *P* value < 0.05 was considered to indicate statistical significance.

### Ethics approval and consent to participate

The study was approved by the Ethics Committee of Shenzhen Children's Hospital (Permission No. 2016020). Informed consent was obtained from all subjects and/or their legal guardians.

## Result

### Study population

**Figure 1:**
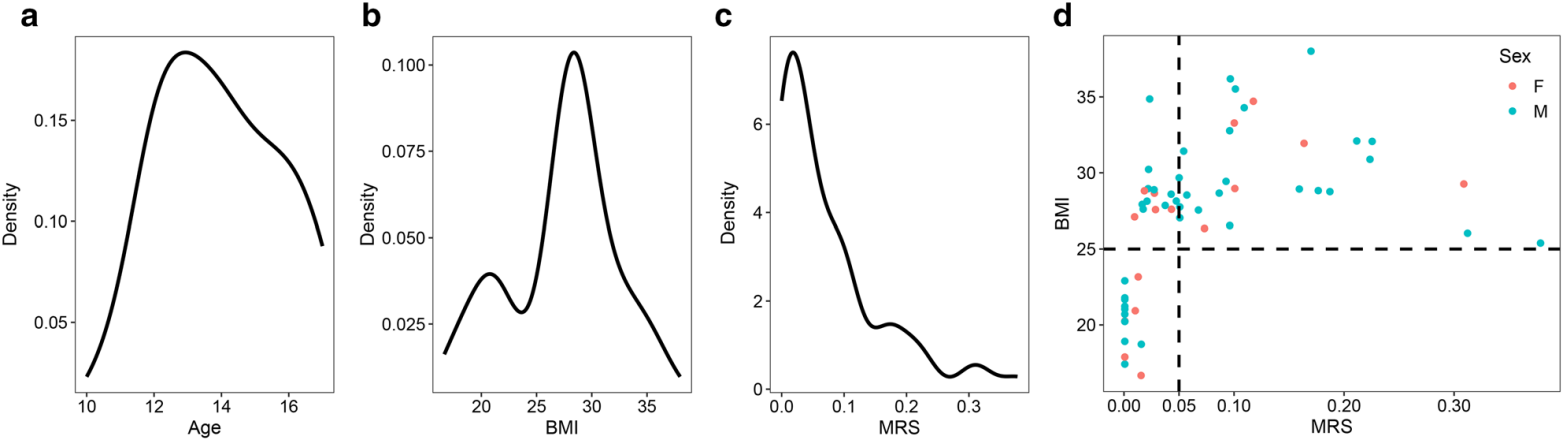
Phenotypes and their distribution in children. (**a**) The age ranges from 10 to 17 years, which is not normally distributed (Shapiro test, *P* < 0.05). (**b**) The BMI which ranges from 16.6 to 38.0, is not normally distributed (Shapiro test, *P* < 0.05). (**c**) The MRS-PDFF ranges from 0 to 0.38, and is not normally distributed as well (Shapiro test, *P* < 0.05). (**d**) The BMI of the children with NAFLD (MRS > 0.05) is > 25, and the BIM and MRS-PDFF have a significant correlation (Spearman’s rho = 0.7). Note: The dotted lines of Fig. 1A, B, C represent the mean.

A total of 58 children, with a median age of 13.7 years (ranging from 10 to 17 years), were studied. BMI ranged from 16.6 to 38.0 kg/m^2^, while the MRS-PDFF ranged from 0 to 0.38. The demographics and quantitative value of MRS-PDFF are shown in Table S1. According to BMI and MRS-PDFF, we divided the participants into BMI groups, including the AW group (n = 43) and Lean group (n = 15) (Table 1A); the MRS groups, including the NAFLD group (n = 27) and the Control group (n = 31) (Table 1B); and the BMI-MRS 3 groups, including NAFLD_AW (AW children with NAFLD) (n = 27), Ctrl_AW (n = 16) (AW children without NAFLD), and Ctrl_Lean (n = 15) (Table 1C). There was no difference in sex or age among those groups (*P* > 0.05). As shown in Fig. 1, age, BMI, and MRS-PDFF were not normally distributed (Shapiro test, *P* < 0.05), while BIM and MRS-PDFF had a significant correlation (Spearman’s rho = 0.7).

**Table 1:** Phenotypic characteristics of BMI groups, MRS groups, and BMI-MRS 3 groups.

	Lean	AW	*p*	
**A** Phenotypic characteristics of BMI groups,				
n	15	43		
Sex = Male(%)	10 (66.7)	32 (74.4)	0.808	
Age (mean (SD))	13.67 (2.02)	14.02 (1.78)	0.522	
BMI (mean (SD))	20.22 (1.94)	29.79 (2.97)	< 0.001	

	Control	NAFLD	*p*	
**B** phenotypic characteristics of MRS groups,				
n	31	27		
Sex = Male (%)	21 (67.7)	21 (77.8)	0.576	
Age (mean (SD))	13.87 (1.65)	14.00 (2.06)	0.792	
BMI (mean (SD))	24.63 (4.72)	30.40 (3.37)	< 0.001	

	Ctrl_Lean	Ctrl_AW	NAFLD_AW	*p*
**C** phenotypic characteristics of BMI-MRS 3 groups				
n	15	16	27	
Sex = Male (%)	10 (66.7)	11 (68.8)	21 (77.8)	0.689
Age (mean (SD))	13.67 (2.02)	14.06 (1.24)	14.00 (2.06)	0.811
BMI (mean (SD))	20.22 (1.94)	28.77 (1.82)	30.40 (3.37)	< 0.001

The BMI between the Ctrl_AW and NAFLD_AW (wilcox test, *P* = 0.12).

### Comparison of gut microbiota ecological diversity between the groups

**Figure 2:**
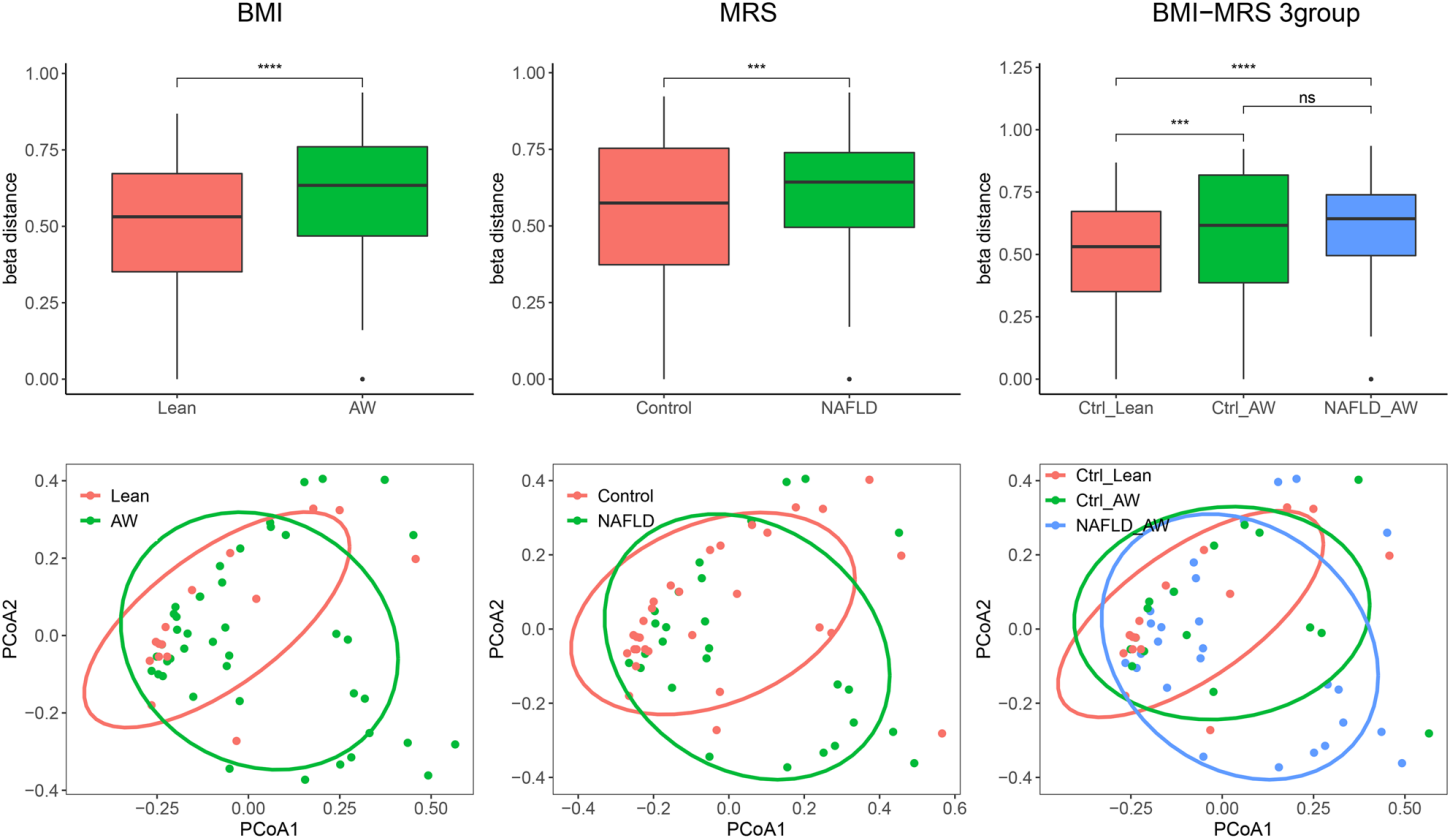
Comparison of gut microbiota ecological diversity between the groups. (Top) The beta diversity is compared between each pair of groups based on the Bray‒Curtis distance at the genus level. The comparisons show that the beta-diversity of the AW group and NAFLD group is higher than that of the healthy lean controls (Wilcoxon rank-sum test, *P* < 0.05)**.** (Bottom) Principal coordinates analysis (PCoA) plot at the genus level also shows consistent findings on the significant difference in microbial composition between the Ctrl-Lean and NAFLD-AW groups (PERMANOVA, Lean vs. AW, *P* = 0.065; Control vs. NAFLD, *P* = 0.121; Ctrl-Lean vs. NAFLD-AW, *P* = 0.039; Control-AW vs. NAFLD-AW, *P* = 0.702). Each point represents a single child. Note: “*”, *P* < 0.05; “**”, *P* < 0.01; “***”, *P* < 0.005.

The Shannon index was used for alpha diversity comparison between the groups at both the genus and species levels. There were no significant differences between any two groups (Wilcoxon rank-sum test, *P* > 0.05) (Supplementary Figure S1). The beta diversity was compared between each pair of groups based on the Bray‒Curtis distance at the genus level. The comparisons showed that the beta-diversity of the AW group and NAFLD group was higher than that of the healthy lean controls (Wilcoxon rank-sum test, *P* < 0.05) (Fig. 2). The PCoA plot at the genus level also showed the consistent findings of a significant difference in microbial composition between the Ctrl-Lean and NAFLD-AW groups (PERMANOVA, Lean vs. AW, *P* = 0.065; Control vs. NAFLD, *P* = 0.121; Ctrl-Lean vs. NAFLD-AW, *P* = 0.039; Control-AW vs. NAFLD-AW, *P* = 0.702) (Fig. 2).

### Taxonomy comparison of the gut microbiota at the genus and species levels

**Figure 3:**
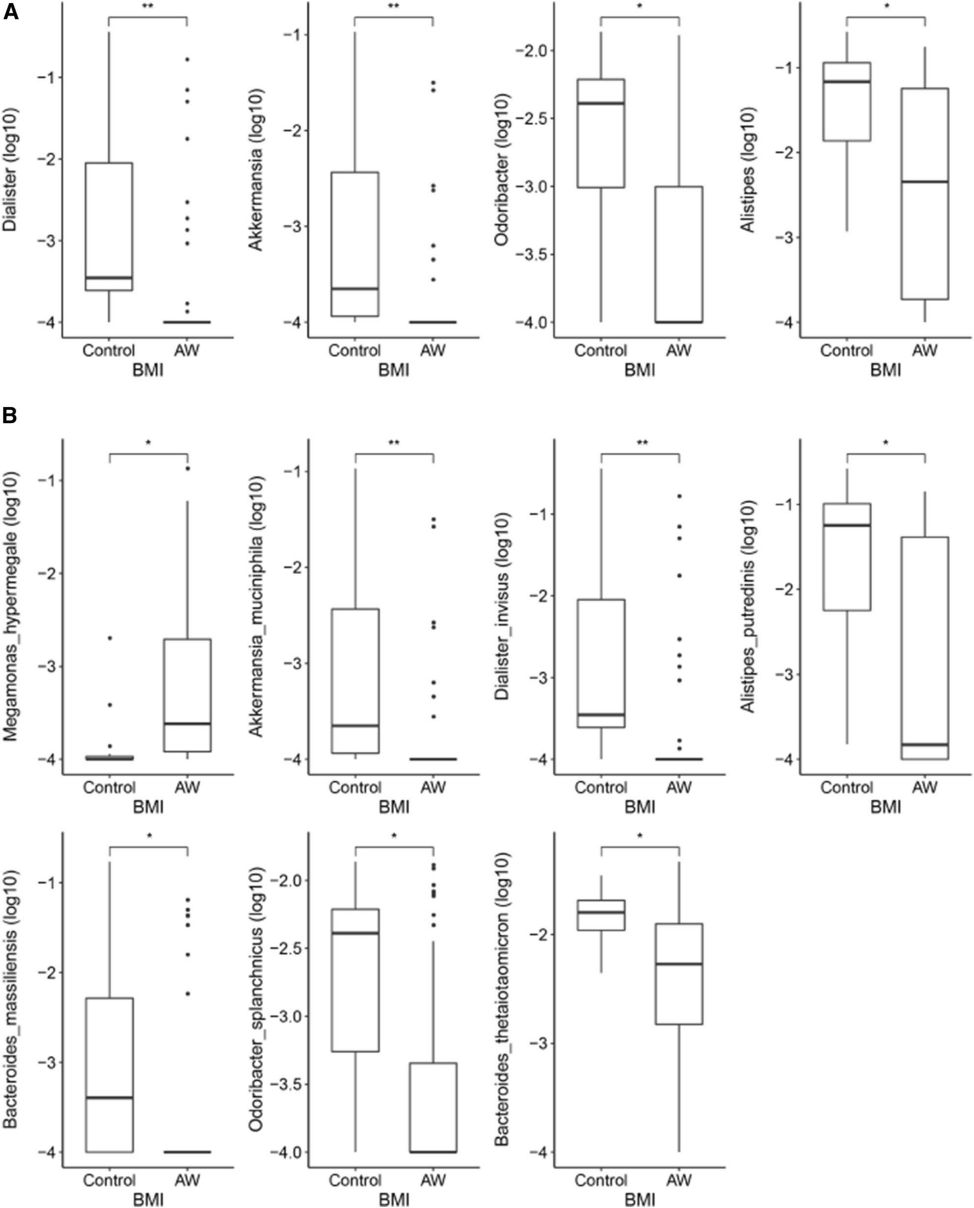
Comparison of gut microbial compositional features at the genus and species levels between the BMI groups using the Wilcoxon rank-sum test. (**A**) At the genus level, *Dialister*, *Akkermansia*, *Odoribacter*, and *Alistipes* exhibit a significant decrease in AW children compared with the Lean children. (**B**) At the species level, *Megamonas hypermegale* is increased in the AW group, while *Akkermansia muciniphila*, *Dialister invisus*, *Alistipes putredinis*, *Bacteroides massiliensis*, *Odoribacter splanchnicus*, and *Bacteroides thetaiotaomicron* are decreased in AW children compared to the Lean group. Note: *, BH-adjusted *P* < 0.05; **, BH-adjusted *P* < 0.01; ***, BH-adjusted *P* < 0.005.

The relative abundance profiles from MetaPhlAn2 were used. A comparison of gut microbial compositional features at the genus and species levels between the groups (Wilcoxon rank-sum test, *P* < 0.05) are detailed in Table S5 and Table S6. As shown in Fig. 3A, in BMI groups within the genus level, *Dialister*, *Akkermansia*, *Odoribacter*, and *Alistipes* exhibited a significant decrease in AW children compared with Lean children. At the species level, *Megamonas hypermegale* was increased in the AW group, while *Akkermansia muciniphila*, *Dialister invisus*, *Alistipes putredinis*, *Bacteroides massiliensis*, *Odoribacter splanchnicus*, and *Bacteroides thetaiotaomicron* were decreased in AW children compared to the Lean group (Fig. 3B).

**Figure 4:**
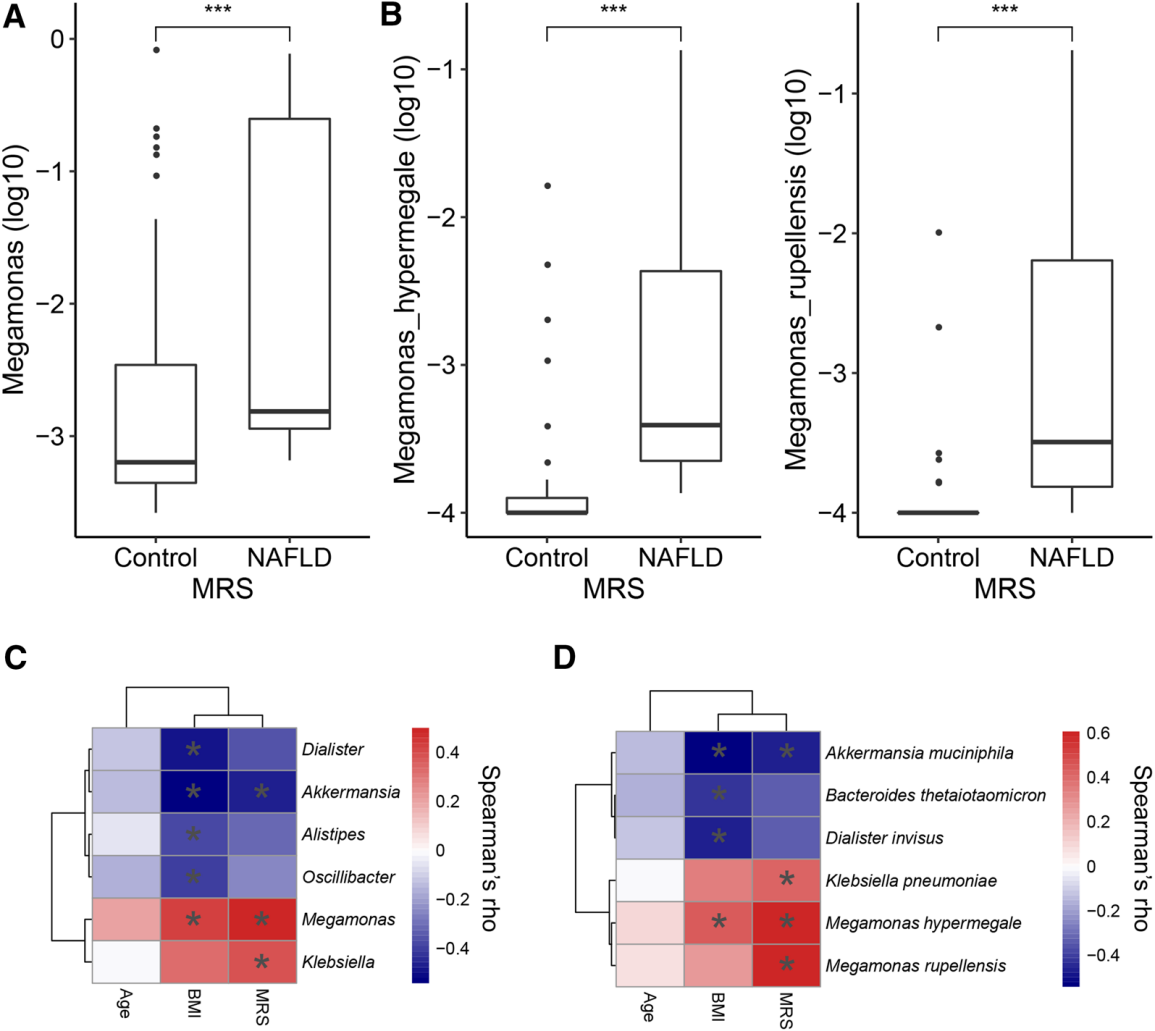
Comparison of gut microbial compositional features at the genus and species levels between the MRS groups using the Wilcoxon rank-sum test. (**A**) The genus *Megamonas* is increased in the NAFLD group compared with the control group. (**B**) The species *Megamonas hypermegale* and *Megamonas rupellensis* are increased in the NAFLD group compared with the Control group. (**C**) Correlations between genus, age, BMI and MRS using Spearman's rank correlation. While the relative abundance of Megamonas is positively associated with BMI and MRS, the abundances of *Dialister*, *Akkermansia*, *Odoribacter*, and Alistipes are negatively correlated with BMI. (**D**) Correlations between species, age, BMI and MRS using Spearman's rank correlation. While the relative abundance of *Megamonas hypermegale* is positively associated with BMI and MRS, that of *Megamonas rupellensis* is significantly and positively correlated with MRS, but not with BMI. On the other hand, *Akkermansia muciniphila*, *Bacteroides thetaiotaomicron* and *Dialister invisus* are negatively correlated with BMI. Note: *, BH-adjusted *P* < 0.05; **, BH-adjusted *P* < 0.01; ***, BH-adjusted *P* < 0.005.

Between the MRS groups, the genus *Megamonas* and the species of *Megamonas hypermegale* and *Megamonas rupellensis* were increased in the NAFLD group compared with the control group, as shown in Fig. 4A and B. Correlations between genus, age, BMI and MRS were determined using Spearman's rank correlation. While the relative abundance of Megamonas was positively associated with BMI and MRS, the abundances of *Dialister*, *Akkermansia*, *Odoribacter*, and Alistipes were negatively correlated with BMI (Fig. 4C). Correlations between species, age, BMI and MRS were determined using Spearman's rank correlation. While the relative abundance of *Megamonas hypermegale* was positively associated with BMI and MRS, that of *Megamonas rupellensis* was significantly and positively correlated with MRS but not with BMI. On the other hand, *Akkermansia muciniphila*, *Bacteroides thetaiotaomicron* and *Dialister invisus* were negatively correlated with BMI (Fig. 4D).

**Figure 5:**
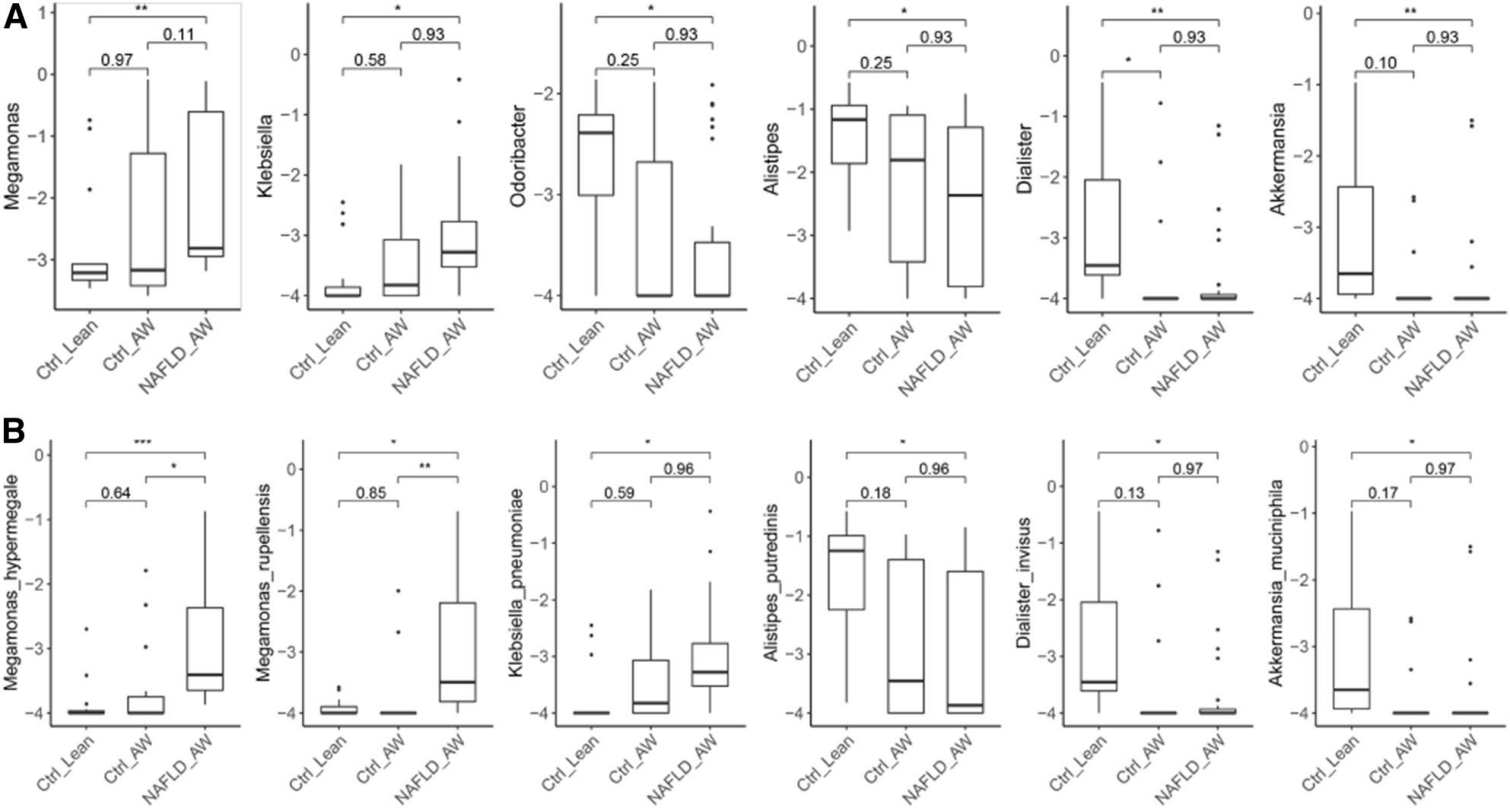
Comparison of gut microbial compositional features at the genus and species levels between the BMI-MRS 3 groups using the Wilcoxon rank-sum test. (**A**) The genus *Megamonas* is enriched in the NAFLD_AW group, while *Odoribacter*, *Alistipes*, *Dialister*, and *Akkermansia* are depleted compared with the Ctrl_Lean or Ctrl_AW group at the genus level. (**B**) *Megamonas hypermegale* and *Megamonas rupellensis* exhibit a significant increase in NAFLD_AW children when compared with the Ctrl_Lean or Ctrl_AW group at the species level. Note: *, BH-adjusted *P* < 0.05; **, BH-adjusted *P* < 0.01; ***, BH-adjusted *P* < 0.005.

Among the BMI-MRS 3 groups, the genus *Megamonas* was enriched in the NAFLD_AW group, while *Odoribacter*, *Alistipes*, *Dialister*, and *Akkermansia* were depleted compared with the Ctrl_Lean or Ctrl_AW groups at the genus level (Fig. 5A). *Megamonas hypermegale* and *Megamonas rupellensis* exhibited a significant increase in NAFLD_AW children compared with the Ctrl_Lean or the Ctrl_AW group at the species level (Fig. 5B). In other words, *Megamonas hypermegale* and *Megamonas rupellensis* were abundant in obese children with NAFLD but not in obese children without NAFLD.

### Pathways of the gut microbiota

**Figure 6:**
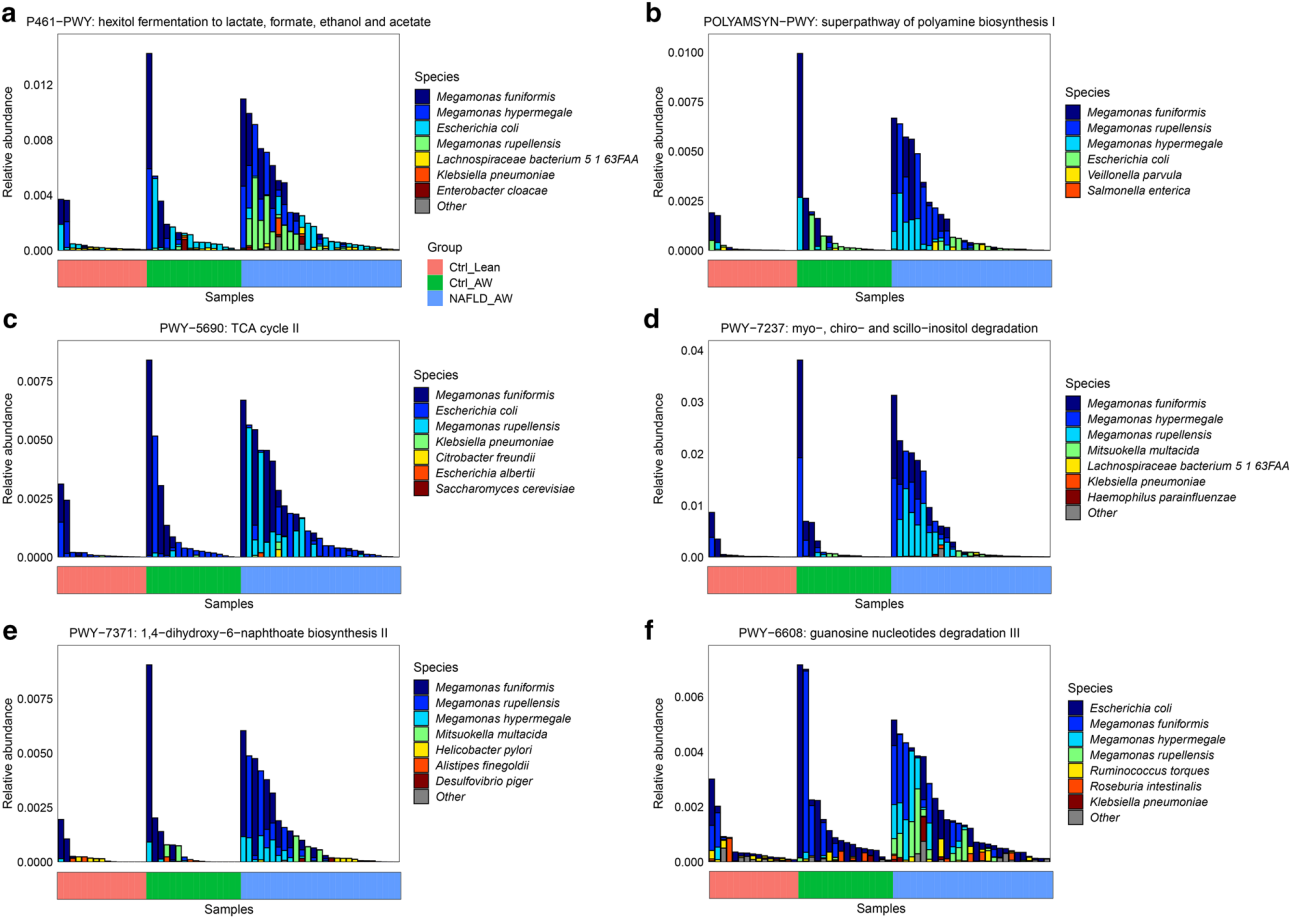
Comparison of the gut microbial pathways contributed by the genus *Megamonas* between the BMI-MRS 3 groups from the HUMAnN2 profiles. (**a**) The pathway of P461-PWY between those groups. (**b**) The pathway of POLYAMSYN-PWY between those groups. (**c**) The pathway of PWY-5690 between those groups. (**d**) The pathway of PWY-7237 between those groups. (**e**) The pathway of PWY-7371 between those groups. (**f**) The pathway of PWY-6608 between those groups. Compared with the Ctrl_Lean (healthy children), the pathways contributed by the genus *Megamonas* are significantly increased in the NAFLD-AW group.

A comparison of gut microbial pathways between the groups is detailed in Table S8. Compared with the Ctrl_Lean (healthy children), the pathways (P461-PWY, POLYAMSYN-PWY, PWY-5690, PWY-7237, PWY-7371, and PWY-6608) contributed by the genus *Megamonas* were significantly increased in the NAFLD-AW group (Fig. 6).

## Discussion

In the current study, we assessed the gut microbiota of 27 obese children with NAFLD, 16 obese children without NAFLD, and 15 healthy controls. We analysed the genus and species levels of the gut microbiota in those groups and their corresponding functional pathways. The BIM and MRS-PDFF were significantly correlated; that is, the BMI and MRS-PDFF values were statistically higher in the NAFLD group, suggesting that obesity and/or hepatic steatosis could be taken as a characteristic of our NAFLD subjects. However, previous studies reported that the gut microbial imbalance in obese and nonobese NAFLD patients was similar, and nonobese NAFLD patients still had a BMI value statistically higher than the controls^20,21^. In this study, the relative abundance of *Megamonas* was positively associated with BMI and MRS, abundances of *Dialister*, *Akkermansia*, *Odoribacter*, and *Alistipes* were negatively correlated with BMI. At the species level, the relative abundance of *Megamonas hypermegale* was positively associated with BMI and MRS, and that of *Megamonas rupellensis* was significantly and positively correlated with MRS, but not with BMI.

Simultaneously, we identified significant differences in which the beta-diversity of NAFLD children was higher than that of healthy children, which was consistent with previous studies^12^. We found that *Dialister*, *Akkermansia*, *Odoribacter*, and *Alistipes* exhibited a significant decrease in AW (obese) children at the genus level compared with Lean (healthy) children. However, the four genera stated above were also decreased in AW (obese) children with NAFLD compared with controls (a combination of obese children without NAFLD and healthy children). Previous studies only reported that one or two of them (*Dialister*, *Akkermansia*, Odoribacter*, Alistipes*) decreased in obese children, adults, or rats with NAFLD^21–24^. Data suggest that the four genera might be decreased at the genus level of the gut microbiota in children with NAFLD.

However, at the species level, *Megamonas hypermegale* was increased in AW (obese) children, while *Akkermansia muciniphila*, *Dialister invisus*, *Alistipes putredinis*, *Bacteroides massiliensis*, *Odoribacter splanchnicus*, and *Bacteroides thetaiotaomicron* were decreased, compared to the healthy controls. However, other studies reported that one or two of them (*Akkermansia muciniphila*, *Alistipes putredinis*, *Bacteroides thetaiotaomicron*) decreased in obese children, adults, or rats with NAFLD^21,24–27^. Can these bacteria supplement weight loss? These can be further studied in the future.

In addition, we found that the genus *Megamonas* was increased in the NAFLD group, compared with the control group, and it was also enriched in the NAFLD_AW group, compared with the Ctrl_Lean and Ctrl_AW groups. Only one study showed that it was significantly more abundant in obese children than in normal-weight children by 16S rRNA gene sequencing^28^. Another study in adults also found that *Megamonas* increased significantly in people with obesity^29^. However, these research did not examine whether the obese children had fatty livers. Data suggest that the genus *Megamonas* might be increased at the genus level of the gut microbiota in obese children with NAFLD but not in non obese children with NAFLD. A study in adults showed that *Megamonas* was associated with NAFLD alone, with no type 2 diabetes mellitus^30^. Another diabetes study found that *Megamonas* was enriched in the diabetes patient with normal glucose tolerance than the newly diagnosed type 2 diabetes mellitus^31^. The above studies suggest that *Megamonas* is related to human metabolic diseases.

Meanwhile, the species of *Megamonas hypermegale* and *Megamonas rupellensis* were increased in the NAFLD group compared with the Control group, while they also exhibited a significantly higher abundance in NAFLD_AW children compared with the Ctrl_Lean and Ctrl_AW groups at the species level. In other words, *Megamonas hypermegale* and *Megamonas rupellensis* were abundant in obese children with NAFLD but not in obese children without NAFLD, which had not been reported before. One study found that *Megamonas hypermegale* and *Megamonas funiformis* were significantly related to fibrosis severity in non-obese NAFLD than in obese NAFLD in adults^32^. Their study found that *Megamonas hypermegale* was highly associated with fibrosis in non-obese NAFLD, but was abundant in obese NAFLD in our study. Unlike them, our study was with children, theirs was with adults, and they studied fibrosis severity.

Furthermore, the pathways contributed by the genus *Megamonas* were significantly increased in the NAFLD-AW group, compared with healthy controls. In these six pathways, P461-PWY is responsible for the fermentation of hhexitol to lactic acid, formic acid, ethanol and acetic acid; POLYAMSYN-PWY is used for amine and polyamine biosynthesis; PWY-5690 is responsible for the production of precursor metabolites and energy; PWY-7237 is involved in myo-inositol degradation; PWY-7371 is involved in the biosynthesis of 1,4-dihydroxy-6- naphthoate; and PWY-6608 is responsible for guanosine nucleotide degradation^33^. All of these six pathways were enriched in adults with metabolic syndrome^33^. Five of them including POLYAMSYN-PWY, PWY-5690, PWY-6608, PWY-7237 and PWY-7371 had no studies related to NAFLD. Previous study has shown that the production of acetic acid and propionic acid by *Magnomonas* was a substrate for adipogenesis and cholesterol formation in rodents^34^. More lipogenesis and cholesterol accumulation may lead to abnormal liver function^33^. Other studies have reported that the metabolic pathway of P461-PWY that was contributed by the genus *Megamonas* or *Megamonas hypermegale* and *Megamonas rupellensis* could produce acetate, which promoted the accumulation of triglycerides and finally promoted the development of NAFLD^35,36^. Therefore, the data suggest that the NAFLD status and/or diet associated with NAFLD patients leads to the enrichment of the genus *Megamonas* or *Megamonas* species.

There were two limitations in this study. One of the limitation was that no detailed food frequency questionnaires were included to account for the dietary effect on the observed differences in the gut microbiota between cases and controls. The other limitation was that our subjects did not have non obese NAFLD.

## Conclusion

We investigated the gut microbiota in a pediatric cohort with and without NAFLD. Future studies concerning gut microbiota and NAFLD might use BMI and MRS-PDFF as two suitable clinical indexes. We identified significant differences in which the beta-diversity of NAFLD children was higher than that of healthy children. Collectively, we found that the genus *Megamonas* was enriched at the genus level in children with NAFLD, while *Megamonas hypermegale* and *Megamonas rupellensis* were enriched at the species level in children with NAFLD; the latter two species were not abundant in obese children without NAFLD. The metabolic pathway of P461-PWY contributed by the genus *Megamonas* or *Megamonas hypermegale* and *Megamonas rupellensis* could produce acetate, which promoted the accumulation of triglycerides and finally promoted the development of NAFLD. Therefore, the NAFLD status and/or diet associated with NAFLD patients may lead to the enrichment of the genus Megamonas or Megamonas species.

## Supplementary Information

Supplementary Information 1.

Supplementary Information 2.

Supplementary Information 3.

## Data Availability

The data availability link for reviewerscan be found in the followinglink (https://dataview.ncbi.nlm.nih.gov/object/PRJNA890867?reviewer=b35h43p8565aod7s3brruvdtnt). The datasets generated for this study can be found in the Sequence Read Archive (https://www.ncbi.nlm.nih.gov/sra) under Bioproject PRJNA890867and will be released upon the publication of this article.

## Abbreviations

NAFLD: Nonalcoholic fatty liver disease
AW: Abnormal weight
MRS: Magnetic resonance spectroscopy
MRS-PDFF: Magnetic resonance spectroscopy proton density fat fraction
BMI: Body mass index
NASH: Nonalcoholic steatohepatitis
PCoA: Principal coordinates analysis
