# Supplementary figures and images for: The relationship of Megamonas species with nonalcoholic fatty liver disease in children and adolescents revealed by metagenomics of gut microbiota
Source: Sci Rep. 2022 Dec 20;12:22001. doi: 10.1038/s41598-022-25140-2 (PMC9767906; doi:10.1038/s41598-022-25140-2)

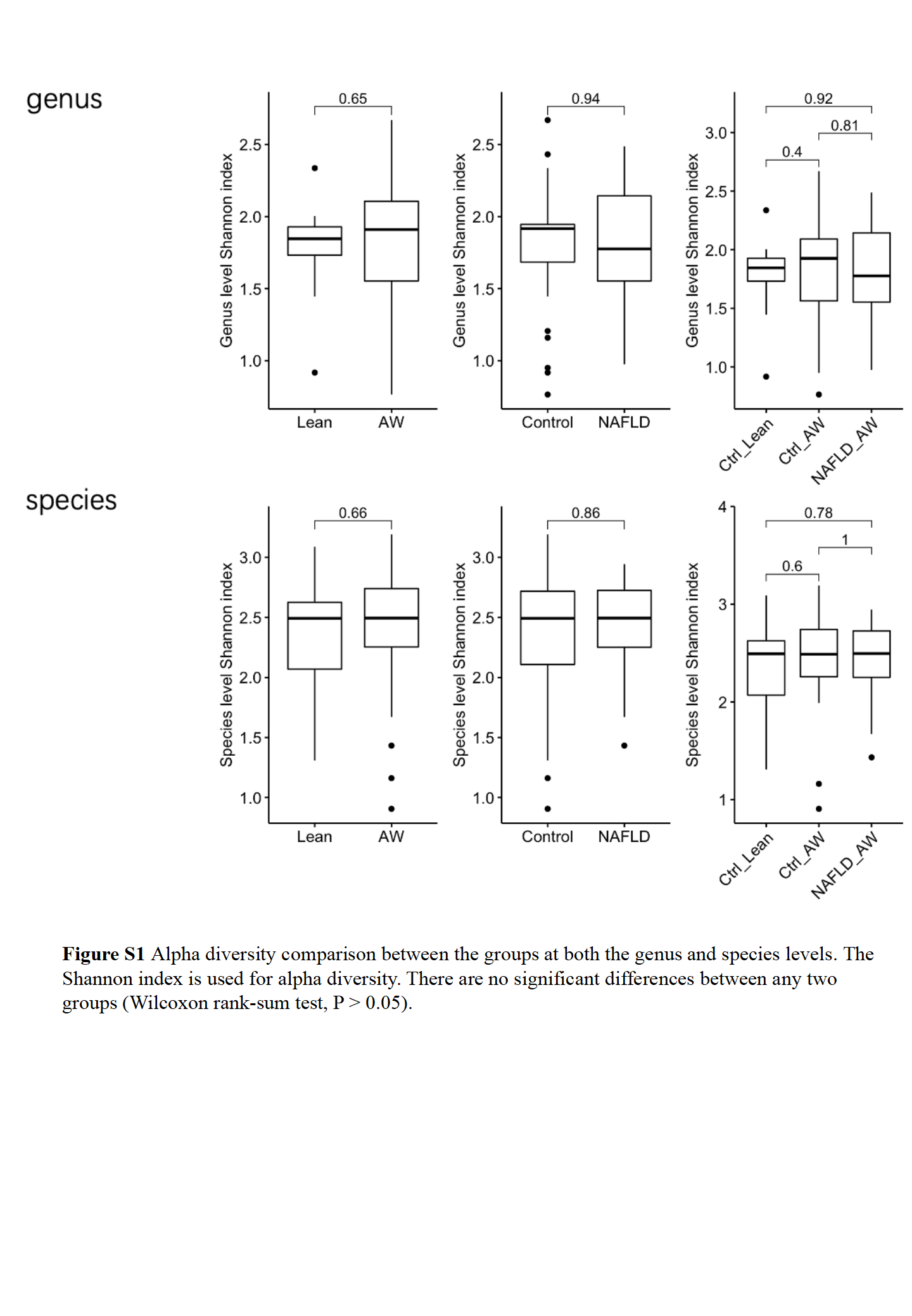

Supplement: Supplementary file 1 — Supplementary Information 1. [file 41598_2022_25140_MOESM1_ESM.tif]
