# Supplementary material for: The relationship of Megamonas species with nonalcoholic fatty liver disease in children and adolescents revealed by metagenomics of gut microbiota
Source: Sci Rep. 2022 Dec 20;12:22001. doi: 10.1038/s41598-022-25140-2 (PMC9767906; doi:10.1038/s41598-022-25140-2)
Supplement: Supplementary file 3 — Supplementary Information 3. [file 41598_2022_25140_MOESM3_ESM.docx]

**Supplementary figure**

**Figure S1** Alpha diversity comparison between the groups at both the genus and species levels. The Shannon index is used for alpha diversity. There are no significant differences between any two groups (Wilcoxon rank-sum test, P > 0.05).

**Supplementary tables**

**Table S1** Characteristics of the study cohort.

**Table S2** Statistics of sequencing data.

**Table S3** Microbial profiling in children (at the genus level).

**Table S4** Microbial profiling in children (at the species level).

**Table S5** Comparison of gut microbial compositional features at the genus level between the groups (Wilcoxon rank-sum test).

**Table S6** Comparison of gut microbial compositional features at the species level between the groups (Wilcoxon rank-sum test).

**Table S7** Microbial pathway profiling in children.

**Table S8** Comparison of gut microbial pathways between the groups (Wilcoxon rank-sum test).
